# Fulminant pancreatic encephalopathy with brain edema and herniation in severe hypertriglyceridemia-induced pancreatitis: a case report

**DOI:** 10.3389/fmed.2026.1968843

**Published:** 2026-10-28

**Authors:** Nivine Aboudargham, Nour El Akach, Nada Hamieh, Aya Rammal, Kassem Barada, Imad Bouakl

**Affiliations:** 1 Division of Pulmonary and Critical Care, Department of Internal Medicine, American University of Beirut, Beirut, Lebanon; 2 Division of Gastroenterology and Hepatology, Department of Internal Medicine, American University of Beirut, Beirut, Lebanon; 3 Department of Internal Medicine, American University of Beirut, Beirut, Lebanon

**Keywords:** acute pancreatitis, brain edema, brain herniation, case report, hypertriglyceridemia, pancreatic encephalopathy

## Abstract

Pancreatic encephalopathy (PE) is a rare but potentially devastating neurological complication of severe acute pancreatitis. Its manifestations range from confusion and agitation to seizures, coma, and cerebral edema, often making the diagnosis challenging due to its overlap with other causes of encephalopathy. We report a 23-year-old woman with hypertriglyceridemia-induced acute pancreatitis who rapidly progressed to severe acute pancreatitis with multiorgan failure. Within 48 h of admission, she developed agitation, seizure-like activity, and coma. Neuroimaging demonstrated diffuse cerebral and cerebellar edema, features of elevated intracranial pressure, cerebellar tonsillar herniation, and extensive restricted diffusion. After evaluation of alternative etiologies, the neurological deterioration was attributed to PE. Despite aggressive medical management, including insulin therapy, renal replacement therapy, mechanical ventilation, and hyperosmolar therapy, she did not show any signs of neurological recovery. This case demonstrates that PE may rarely progress to malignant cerebral edema and tonsillar herniation. New neuropsychiatric manifestations in patients with severe acute pancreatitis should prompt urgent neurological assessment and early neuroimaging whenever clinically feasible.

## 1 Introduction

Severe acute pancreatitis (SAP) occurs in almost one third of cases of acute pancreatitis (AP) (1). It is defined by the American College of Gastroenterology as persistent organ failure lasting more than 48 h, which include shock, renal failure, respiratory insufficiency or GI bleeding (2) The only neurological complication of AP described in literature is in the form of encephalopathy. Pancreatic encephalopathy (PE) is a rare complication of SAP that was first described in 1941 (3). PE remains poorly understood with respect to its underlying pathophysiology, diagnostic criteria and management strategy. It is described in the literature as the onset of many neuropsychiatric symptoms that usually occur in the first two weeks of pancreatitis in the form of altered mental status, psychomotor agitation, visual or auditory hallucinations, seizures, focal neurologic deficits, and even coma. These symptoms are usually remitting relapsing in nature (4). PE is classified as either early or late in the course of SAP. Early PE occurs in the first 2 weeks of diagnosis and is part of the multiorgan failure. While later, PE is mostly related to prolonged fasting, vomiting and the loss of Vitamin B1 known as Wernicke's encephalopathy (5, 6).

The prevalence and incidence of PE remain highly variable and are not well established. Reported incidence rates among patients with AP range from 0.04% to up to 11%, depending on the studied population (7, 8). This wide range of incidence underscores the diagnostic challenge of PE where no clear definition is established by current guidelines. Differentiating PE from other causes of encephalopathies is particularly difficult because of its nonspecific clinical features and the necessity of excluding other potential overlapping etiologies (9).

We present a rare case of severe hypertriglyceridemia induced AP in a young female complicated by rapidly progressive metabolic encephalopathy, diffuse malignant brain edema and herniation.

## 2 Case description

A 23-year-old woman, known to have insulin resistance on metformin, presented to the emergency department with severe upper abdominal pain, nausea and vomiting of one-day duration. She denied alcohol use, recent medication changes, supplement intake, fever, or recent illness. Family history was significant for familial hypertriglyceridemia in her mother and aunt.

On presentation, she was alert and oriented but appeared distressed because of pain. Vital signs showed blood pressure of 157/103 mmHg, heart rate of 103 beats/min, normal oxygen saturation, and no fever. Physical examination revealed marked epigastric tenderness without guarding or rebound tenderness. Her body mass index was 40.9 kg/m^2^.

Initial laboratory evaluation showed mild anemia with hemoglobin of 11.9 g/dL, white blood cell count of 10,900/µL, bicarbonate of 13 mmol/L, high anion gap and normal pH of 7.41, creatinine of 0.74 mg/dL, blood urea nitrogen of 9 mg/dL, glucose of 127 mg/dL, and normal serum calcium of 8.9 mg/dL. Liver function tests were unremarkable. Serum triglycerides were markedly elevated at 7,533 mg/dL, lipase was 292 U/L, and C-reactive protein was 37.3 mg/L. Computed tomography of the abdomen and pelvis showed mild acute interstitial pancreatitis with peripancreatic inflammatory changes, without gallstones, biliary obstruction, pancreatic necrosis, or peripancreatic fluid collections.

She was diagnosed with hypertriglyceridemia-induced AP with an initial BISAP score of 1 and was admitted to the intensive care unit for close monitoring. Initial management included bowel rest, intravenous hydration with 1L of normal saline followed by maintenance lactated ringer at rate of 1.5 mL/kg/h, analgesia with paracetamol and morphine, and endocrinology-guided intravenous insulin infusion at 10 U/h.

Ten hours after admission, her condition deteriorated with worsening sinus tachycardia reaching 160 beats/min and tachypnea up to 35 breaths/min followed by high-grade fever 20 h post admission. Repeat laboratory testing showed hemoconcentration, with hemoglobin increasing to 15.7 g/dL, acute kidney injury with creatinine rising to 1.44 mg/dL, persistent metabolic acidosis, and severe hypocalcemia with calcium of 6 mg/dL. Additional 3L of intravenous fluids and 2 g of calcium gluconate were given. Blood cultures were withdrawn and empiric piperacillin-tazobactam was initiated, knowing that her chest Xray and urinalysis were negative. At this time, urine output was maintained at 100–150 mL/hr.

36 h after admission, the patient developed acute neuropsychiatric manifestations characterized by agitation, disorientation, and delusional behavior, with the belief that she was pregnant. She was started on low dose Dexmedetomidine for agitation. She then developed seizure-like activity with upward and rightward eye deviation, tonic posturing, and unresponsiveness. Serum calcium around the time of the seizure reached 5.6 mg/dL (corrected calcium=6.9 mg/dl) so it was immediately replaced. The episode was diagnosed as provoked seizure for which IV diazepam was administered followed by loading and maintenance doses of levetiracetam. At that time, brainstem reflexes, including pupillary and corneal reflexes, remained intact.

Because of persistent unresponsiveness lasting 15 min after seizure-like activity and respiratory failure, she underwent endotracheal intubation for airway protection. She then developed hypotension with systolic blood pressure reaching 80s requiring phenylephrine support. At the time of the initial neurologic deterioration, neuroimaging was considered but deferred because she was hemodynamically unstable for transport, given dual pressor requirement.

Post-intubation laboratory evaluation showed worsening metabolic acidosis with bicarbonate of 8 mmol/L, arterial pH of 7.16 and creatinine of 3 mg/dl. Triglyceride had decreased to 1,419 mg/dL. In the setting of progressive kidney injury, oliguria, and refractory metabolic acidosis, continuous renal replacement therapy was initiated. Her temperature remained persistently elevated at 41 degrees Celsius for which normothermia protocol was initiated. Neurologic examination revealed severely depressed mental status with no motor response to painful stimuli, despite preservation of brainstem reflexes. Triglyceride levels continued to decline, reaching 617 mg/dL.

The next day, her neurologic status deteriorated further with loss of brainstem reflexes. Computed tomography of the brain demonstrated diffuse cortical-subcortical hypodense areas, features of elevated intracranial pressure and tonsillar herniation (Figure 1). Sodium level was ranging between 146 and 148 at this time and has been gradually increasing over the first 72 h starting from 133. Hyperosmolar therapy with mannitol and hypertonic saline was initiated. Magnetic resonance imaging showed diffuse cerebral and cerebellar edema, cerebellar herniation and extensive restricted diffusion involving the cerebral and cerebellar white matter. Magnetic resonance angiography demonstrated absent flow-related enhancement within the cervical and intracranial arterial circulation (Figure 2). Electroencephalography showed a severely suppressed background, with no identifiable cerebral activity above noise at a sensitivity of 70 µV/cm.

**Figure 1:**
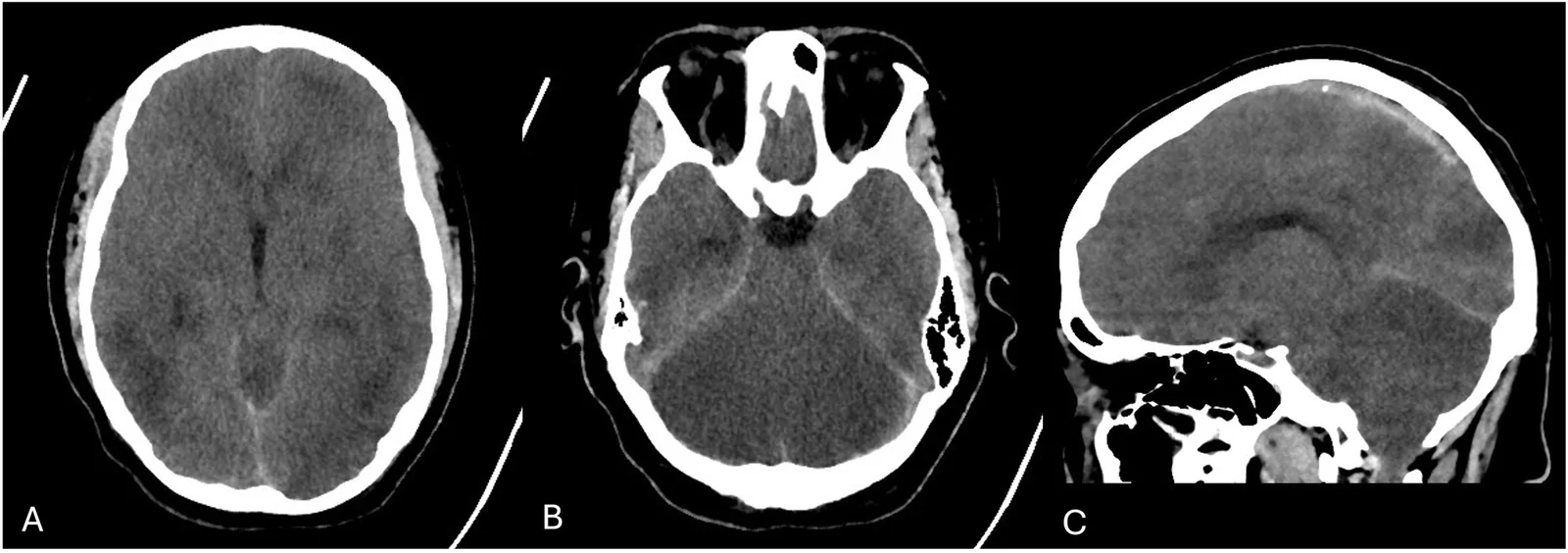
Computed tomography of the brain showing diffuse bilateral cortical subcortical hypodense areas associated with sulcal effacement **(A)** there is diffuse loss of the grey-white differentiation at the level of the cerebellum with significant mass effect on the fourth ventricle **(B)** associated with secondary tonsillar herniation **(C)**.

**Figure 2:**
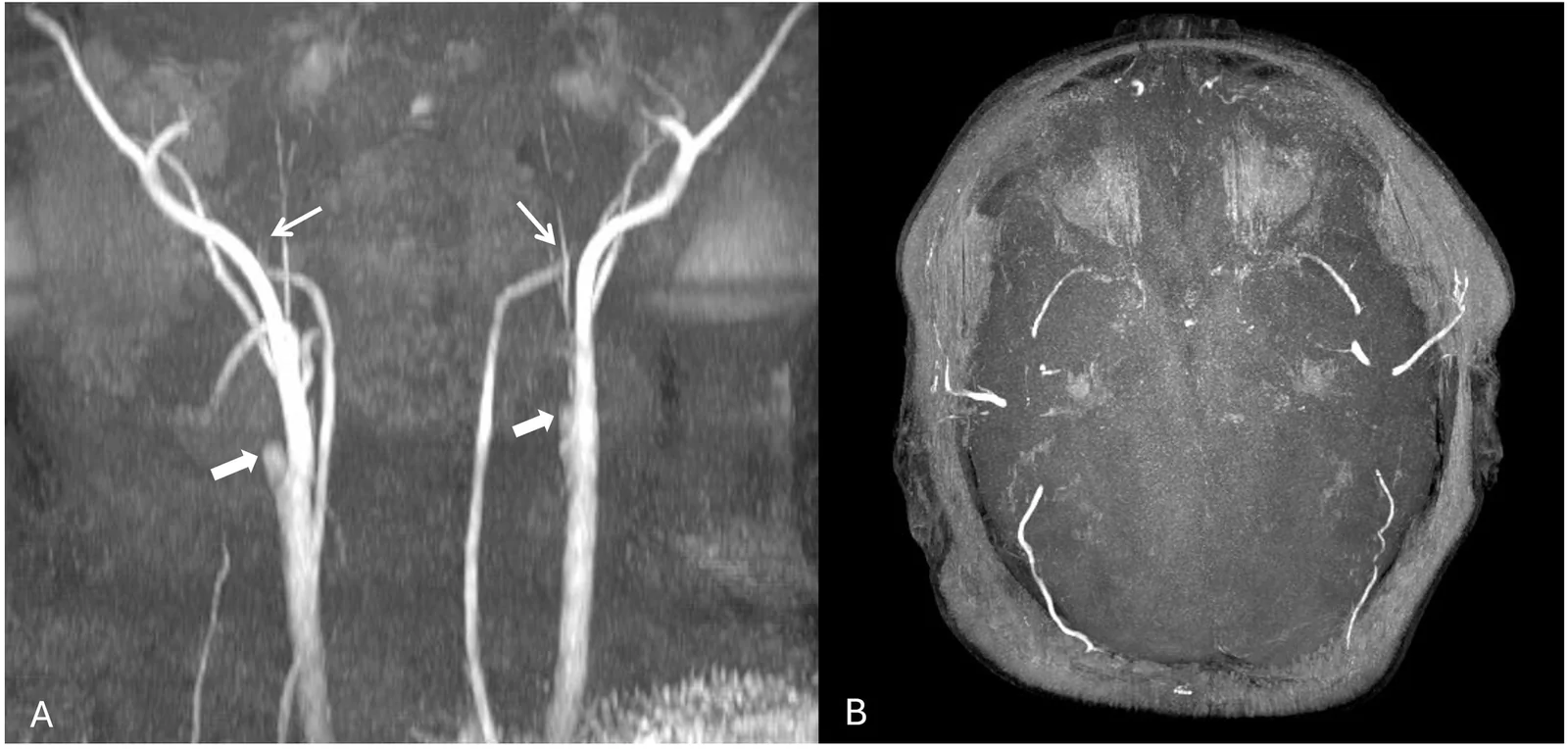
MRA of the brain. **(A)** Absense of flow related enhancement in the internal carotid artery approximately 8mm after its takeoff (block arrow) and in the vertebral arteries at their V3 segment (line arrow). **(B)** MIP image showing absence of flow related enhancement in the major intracranial vessels. MRA: Magnetic resonance angiography. MIP: Minimal intensity projection.

Given that the neurologic decline paralleled the worsening systemic inflammatory response and multiorgan failure associated with pancreatitis, PE was favored as the primary diagnosis. Because PE remains a diagnosis of exclusion, alternative contributors were carefully considered. Review of the chart did not reveal any sustained oxygen desaturation or prolonged hypotension, making hypoxia and ischemia unlikely causes.

The patient was managed through a multidisciplinary approach involving the intensive care, neurology, neurosurgery, endocrinology and gastroenterology teams. Given the severity of the clinical and radiologic findings, the consensus was that there was no chance of meaningful functional neurologic recovery. Three months after presentation, the patient remained intubated with no meaningful neurologic recovery Figure 3.

**Figure 3:**
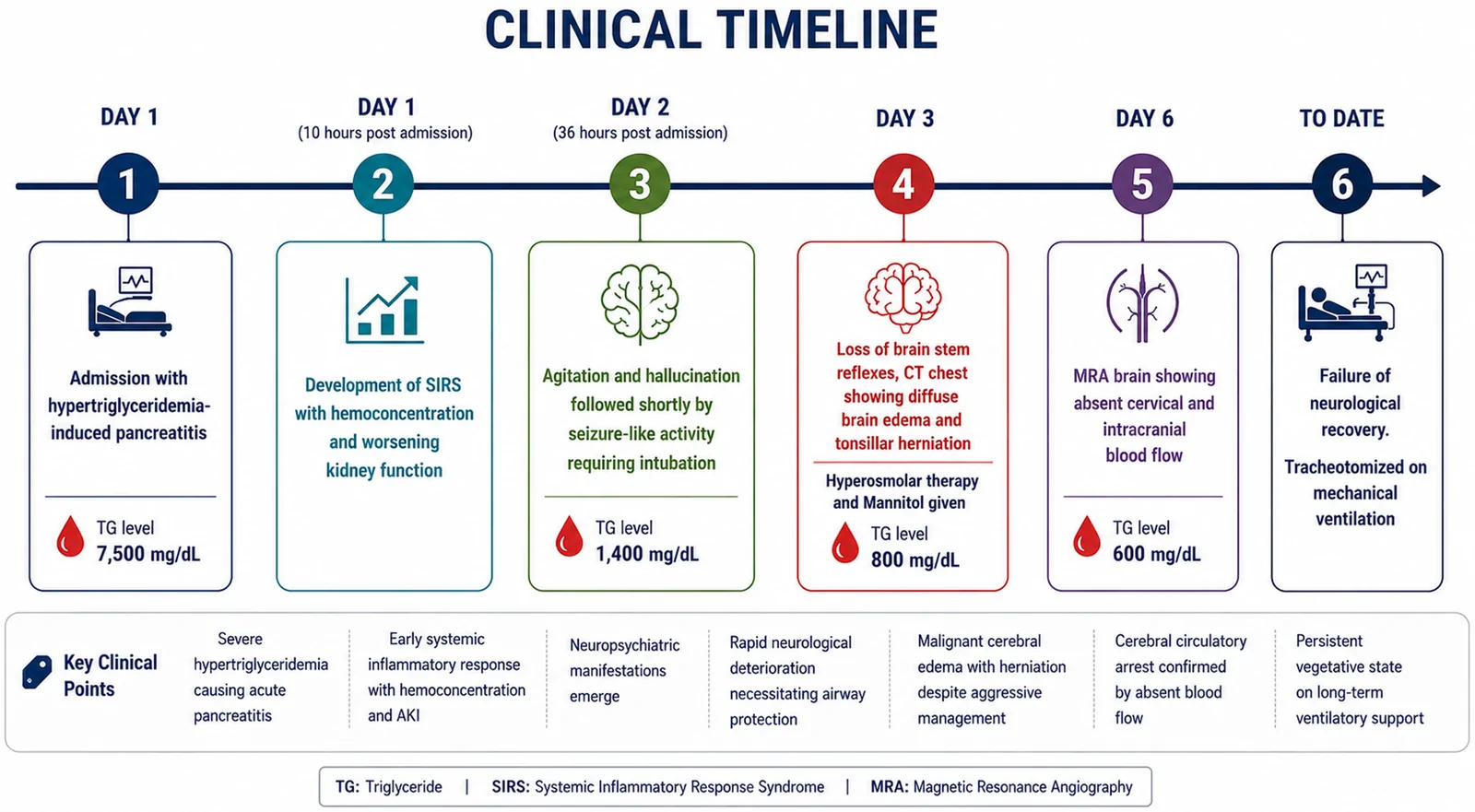
Timeline of relevant events.

## 3 Discussion

PE is an uncommon but potentially devastating neurological complication of SAP with high mortality rates ranging from 43% up to 70% (7, 12). SAP, which develops in approximately one third of patients with AP (1), is considered the major and most consistently reported risk factor for PE (7, 12). However, despite the relatively frequent occurrence of SAP, PE remains infrequently recognized, with only a limited number of well-documented cases reported in the literature. This observation suggests that factors beyond disease severity alone may contribute to the development of PE (7, 12). According to a recent meta-analysis, multiorgan failure and chronic cholecystitis were the most significant reported risk factors for mortality in patients with PE (7).

Although obesity has not been identified as an independent risk factor for PE, substantial evidence demonstrates that obesity, particularly BMI >40 kg/m2, and insulin resistance predispose patients to more SAP, multiorgan failure and higher mortality (13, 14). Because PE predominantly occurs in severe disease, obesity and metabolic syndrome in our patient may have indirectly increased her susceptibility to PE. This hypothesis, however, has not been specifically investigated and warrants further study.

The pathogenesis of PE is complex and remains incompletely understood. Proposed mechanisms include activation of pancreatic enzymes such as trypsin and phospholipase A2, resulting in membrane phospholipids degradation and blood-brain barrier disruption, ultimately causing cytotoxic and vasogenic cerebral edema (15). In addition, the systemic inflammatory response associated with SAP leads to the release of proinflammatory cytokines, including tumor necrosis factor-alpha (TNF-α), interleukin (IL)-1β, IL-6, and IL-10, which further contribute to endothelial dysfunction, increased vascular permeability, and neurological injury. Oxidative stress together with hypoxemia, metabolic derangements, and microvascular injury may all contribute to white matter demyelination and diffuse brain injury (6, 12, 16). More recent evidence supports a complex bidirectional pancreas-brain access, where disruption of the BBB primes the brain for subsequent neuroinflammation and neuro-glial-immune interactions that trigger a self-amplifying cascade and drive disease progression (17).

Recognition of PE is hindered by its nonspecific presentation and the absence of established diagnostic criteria. Neurological manifestations can occur along with several competing conditions commonly encountered during SAP. These include sepsis-associated encephalopathy, hypoxemia, electrolyte and glucose derangements, hepatic or uremic encephalopathy, and cerebrovascular events (9). Although PE has sometimes been considered a manifestation of sepsis-associated encephalopathy, differences in the proposed underlying pathophysiological mechanisms suggest that the two entities may be distinct (6).

In our patient, severe hypocalcemia may have contributed to the seizure through its neuroexcitatory effects. However, it was promptly corrected and it would not be expected to explain the subsequent diffuse cerebral edema and herniation (10, 11). Other possible contributors included kidney injury, metabolic acidosis, sepsis and high-grade fever as well as post-ictal state. However, none was sufficient to explain the catastrophic neurologic course and the development of cerebral edema. Infectious meningoencephalitis was considered less likely in the absence of preceding infectious symptoms and the imaging findings favoring diffuse global injury rather than a focal infectious process. Neurologic deterioration occurred within 48 h of admission, in parallel to the worsening inflammatory response, beginning with agitation, disorientation, and delusional behavior before progressing to seizure-like activity and coma. This temporal pattern is consistent with previous reports demonstrating that PE most commonly develops during the early inflammatory phase of SAP, typically within the first two weeks of illness (4, 6).

To the best of our knowledge, this is the first reported case of brain herniation secondary to PE. Our patient not only exhibited the neuropsychiatric symptoms typically associated with PE but also developed catastrophic cerebral edema leading to cerebellar herniation. A recent national cohort in 2025 reported an incidence of cerebral edema of only 0.04% among patients with AP (8). To our knowledge, the only previously published case of PE associated with cerebral edema related to hypertriglyceridemia-induced pancreatitis was reported by Gill et al (18). Moreover, only three published case reports have clearly documented PE with radiologically confirmed diffuse cerebral edema in the setting of AP (Table 1).

**Table 1:** Published cases of pancreatic encephalopathy with radiologically confirmed diffuse cerebral edema associated with acute pancreatitis (AP).

Study	Etiology of AP	Severity of AP	Brain Imaging Findings	Treatment Measures	Outcome
Gill et al., 2017 (Am J Med) (18)	Hypertriglyceridemia	Severe AP requiring ICU admission	CT brain: diffuse cerebral edema	Intensive supportive care, treatment of hypertriglyceridemia, mechanical ventilation, neurocritical care, and management of severe acute pancreatitis	Survived with neurological improvement
Paras et al., 2026 (Case Reports in Critical Care) (9)	Etiology not definitively established	Severe necrotizing acute pancreatitis complicated by septic shock, ARDS, acute kidney injury, and pancreatic encephalopathy	CT brain: diffuse cerebral hypoattenuation, sulcal effacement, loss of gray-white differentiation, cerebellar edema, scattered microhemorrhages	Mechanical ventilation, aggressive IV fluids, insulin infusion, vasopressors, CRRT, antibiotics, hypertonic saline (5%), antiepileptic therapy, thiamine, enteral nutrition, and supportive neurocritical care	Gradual neurological recovery with complete radiological resolution of cerebral edema; discharged without focal neurological deficits
Minhas et al. 2020 (CHEST) (19)	Probably alcohol induced	Severe acute pancreatitis complicated by ARDS	CT brain: diffuse brain edema and trace subarachnoid hemorrhage	Conservative management consisting of raising and maintaining a serum sodium goal of 150.	Complete recovery without any neurological deficit

The present case shares several features with the report by Gill et al., including severe hypertriglyceridemia, acute pancreatitis, renal failure, diffuse cerebral edema, and neurological deterioration. However, the clinical outcomes differed markedly. In the case described by Gill et al., the reduction in triglyceride levels through insulin therapy and plasmapheresis was associated with radiographic improvement and complete neurological recovery (18). In contrast, our patient experienced progressive neurological decline despite a rapid reduction in triglyceride from 7,533 mg/dL to below 1,500 mg/dL within 48 h followed by subsequent normalization. This observation suggests that once extensive blood-brain barrier disruption, neuroinflammation, and cerebral edema have developed, neurological injury may continue to progress independently of serum triglyceride levels.

The devastating neurological outcome in our patient stands out among the expected recovery mentioned in the literature. High mortality in PE is mainly due to multiorgan failure. However, among survivors, neurological recovery seems to be favorable. Most reported cases of cerebral edema in PE resulted in gradual recovery of clinical and radiological features (9, 18, 19). According to Meng et. al, around 70% of patients with AP who developed PE had improvement in clinical symptoms (7). Ruggieri followed up his patient with remitting and relapsing PE for 7 years and persistent cognitive impairment (20). Our patient developed malignant brain edema with herniation and no chances of recovery within less than 1 week of the onset of SAP. She remains in coma with no neurological recovery three months after admission.

The management of PE remains largely supportive (6). The American college of gastroenterology does not mention encephalopathy as a complication of SAP and no clear management strategy exists (2). The mainstay of treatment in PE remains treating the underlying pancreatitis and inflammation (9). This encompasses early aggressive fluid resuscitation which reduces the rates of mortality and morbidity (4), in addition to correction of metabolic abnormalities, nutritional support, and management of organ failure. Other therapies are being studied including low molecular weight heparin, rosveterol, somatostatin, tumor necrosis factor alpha (6). Our patient received prompt insulin therapy, aggressive intravenous hydration, electrolyte replacement, renal replacement therapy, mechanical ventilation and vasopressor support.

Neuropsychiatric manifestations of PE should be managed promptly. Therapy includes neurotropic, antipsychotic and antiepileptic drugs (7). Most literature consider thiamine/Vitamin B1 as essential part in treatment and prevention of PE (4, 5). In the late phase of PE, B1 deficiency occurs with prolonged fasting, vomiting and increased thiamine utilization. Given its role in the synthesis of neurotransmitters and maintenance of myelin sheath, replenishing thiamine may result in restoration of brain function (6, 9). In our patient, maybe thiamine replacement should have been considered. However, given that the neurological deterioration was too early in the course of pancreatitis, thiamine deficiency was unlikely.

Management of cerebral edema is not studied in PE but rather extrapolated from the management of high intracranial pressure in ischemic strokes, intracranial bleed or traumatic brain injury as per neurocritical care guidelines (9). General measures include head of bed elevation, maintenance of normothermia, avoidance of systemic hypotension, and correction of metabolic disturbances. Hyperosmolar therapy with hypertonic saline or mannitol may be considered to reduce intracranial pressure and cerebral edema. Hypertonic saline is often preferred in critically ill patients because of its more sustained effect on intracranial pressure and lower risk of hypovolemia and renal complications (21). In our patient, normothermia protocol was initiated early in the phase of deterioration followed by hypertonic saline and mannitol once PE was recognized. However, hyperosmolar therapy was withheld shortly after as it was deemed futile based on the extent of brain injury.

## Conclusion

Pancreatic encephalopathy is a rare, poorly defined, and likely underrecognized neurological complication of acute pancreatitis. This case emphasizes that neurological injury can develop early in acute pancreatitis, even in a young patient with an initially low severity score and despite rapid biochemical improvement following standard therapy.

Prompt neurological assessment and early neuroimaging should be considered when feasible, as delayed recognition of evolving cerebral edema may limit therapeutic options. Further research is needed to clarify the mechanisms of PE, identify patients at risk for severe neurological complications, and establish evidence-based strategies for prevention, monitoring, and management.

### Limitations

As in other reported cases of pancreatic encephalopathy, the diagnosis was made largely by exclusion. Although the main alternative causes were considered and discussed above, a definite causal relationship between pancreatitis and neurological deterioration could not be established. Also, the contribution of hypocalcemia, seizure, sepsis and metabolic derangement to neurological deterioration could not be totally and objectively ruled out. Finally, because this is a single case, the findings cannot be used to determine the incidence of this complication, identify generalizable risk factors, or establish a standard treatment approach.

## Patient perspective

Owing to the patient's catastrophic neurological deterioration and unresponsiveness, a patient perspective could not be obtained. The family consented to publication of this case in the hope that it will increase awareness of this rare and devastating complication of acute pancreatitis.

## Data Availability

The original contributions presented in the study are included in the article/Supplementary Material, further inquiries can be directed to the corresponding author.
